# The innate immune signaling component FBXC-58 mediates dietary restriction effects on healthy aging in *Caenorhabditis elegans*

**DOI:** 10.18632/aging.204477

**Published:** 2023-01-06

**Authors:** Jeong-Hoon Hahm, Farida S. Nirmala, Pyeong Geun Choi, Hyo-Deok Seo, Tae Youl Ha, Chang Hwa Jung, Jiyun Ahn

**Affiliations:** 1 Aging and Metabolism Research Group, Korea Food Research Institute, Wanju-gun 55365, South Korea; 2 Department of Food Biotechnology, University of Science and Technology, Daejeon-si 34113, South Korea

**Keywords:** dietary restriction, aging, innate immunity, F-box protein, *Caenorhabditis elegans*

## Abstract

Dietary restriction (DR) is a highly effective and reproducible intervention that prolongs longevity in many organisms. The molecular mechanism of action of DR is tightly connected with the immune system; however, the detailed mechanisms and effective downstream factors of immunity that mediate the beneficial effects of DR on aging remain unknown. Here, to investigate the immune signaling that mediates DR effects, we used *Caenorhabditis elegans*, which has been widely used in research, to understand the underlying molecular mechanisms of aging and immunity. We found that the F-box gene, *fbxc-58*, a regulator of the innate immune response, is a novel mediator of DR effects on extending the health span of *C. elegans*. *fbxc-58* is upregulated by DR and is necessary for DR-induced lifespan extension and physical health improvement in *C. elegans*. Furthermore, through DR, *fbxc-58* prevents disintegration of the mitochondrial network in body wall muscle during aging. We found that *fbxc-58* is a downstream target of the ZIP-2 and PHA-4 transcription factors, the well-known DR mediator, and *fbxc-58* extends longevity in DR through an S6 kinase-dependent pathway. We propose that the novel DR effector, *fbxc-58*, could provide a new mechanistic understanding of the effects of DR on healthy aging and elucidate the signaling mechanisms that link immunity and DR effects with aging.

## INTRODUCTION

The rapidly increasing incidence of various age-related chronic and degenerative diseases, such as sarcopenia [1], in the global elderly population, is a major threat to human healthcare. Thus, a mechanistic understanding of aging and developing intervention measures to combat age-related health complications are of utmost importance in these aged society. The most effective and reproducible aging process intervention to date is dietary restriction (DR), which has shown promising results in increasing longevity and providing a healthy lifestyle in all model organisms tested to date [2]. Moreover, DR is known to be consistent in positively affecting various health-related quality of life attributes in humans [3]. Thus, research into the molecular mechanisms of action of DR is important and relevant.

It has been reported that DR may activate the immune response in animal models, including *Caenorhabditis elegans* [4] and *Drosophila* [5]. Furthermore, DR promotes production or maintenance of naïve T cells in mice and in primates [6, 7]. In humans, mild DR (14% DR) also improved thymopoiesis in thymus, which is important for immune function [8]. However, depending on the circumstance, DR may also increase severity of viral and parasitic infections [9, 10]. Thus, the immune system is tightly connected with DR. Immunity is also associated with aging, and impaired immunity is a key hallmark of aging in many animal species [11]. A recent study showed that, in *C. elegans*, innate immune signaling component is necessary for DR-induced longevity and health improvement [4]. Furthermore, DR enhances the lifespan of *C. elegans* by maintaining the level of p38-ATF-7 (a transcription factor downstream of p38) innate immune response at the level of basal activation [12], suggesting that the immune system plays an important role in the extension of lifespan by DR. However, the detailed mechanisms and effective downstream factors of immunity that mediate the beneficial effects of DR on health span remain unknown.

*Caenorhabditis elegans* is an invaluable animal model for studying aging [13] or age-associated diseases such as immunosenescence [14]. Several studies with *C. elegans* have unraveled various intricate molecular pathways regulating longevity that have been proven to be conserved in higher organisms [13]. A feeding-defective mutant strain, *eat-2*, which mimics DR [15], has been widely used in research to understand the underlying molecular mechanisms of DR-induced lifespan extension. Further, the innate immune system of *C. elegans* is evolutionarily conserved in the animal kingdom Thus, *C. elegans* is used in basic research on the mechanisms of resistance to pathogens [16].

F-box proteins are well-conserved proteins from yeast to humans. They have F-box protein motif which functions as a site for protein-protein interaction. The function of the F-box protein motif has been extensively studied in relation to ubiquitin-mediated proteolysis [17]. The function of F-box proteins in relation to the regulation of aging process or behavior response to pathogen has been reported in *C. elegans* [18, 19]. In this study, we aimed to identify the novel innate immune signaling component that mediates the DR effect, and we found that the F-box protein, FBXC-58, a regulatory component of the innate immune response, is an important downstream effector of DR for extending longevity and improving physical health conditions in *C. elegans*.

## RESULTS

### Screen to uncover the innate immune signal mediating the DR effect

**Figure 1:**
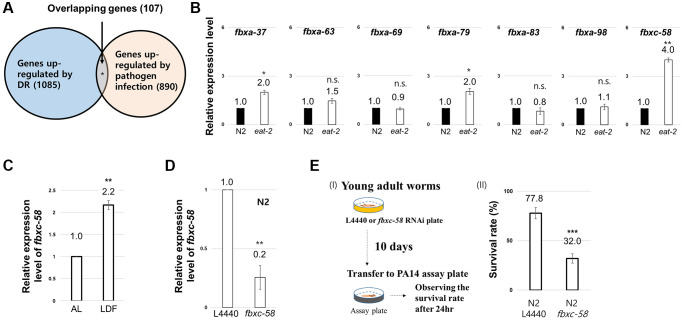
**Screen to uncover the innate immune signal mediating the dietary restriction (DR) effect.** (**A**) Venn diagram depicting the genes overlapping between genes upregulated in DR-worms and upregulated in pathogen infected worms. (**B**) Relative expression level of each F-box gene between wild-type (N2) and *eat-2(ad1116)* mutant strains at day 2 of adulthood. (**C**) Relative expression levels of *fbxc-58* mRNA in N2 on ad libitum (AL) and low density of food (LDF) condition at day 2 of adulthood. (**D**) Relative expression level of *fbxc-58* in L4440 (L4440 RNAi N2) and *fbxc-58* (*fbxc-58* RNAi N2) at day 3 of adulthood. (**E**) (I) Schematic diagram for *Pseudomonas aeruginosa* (PA14) infection assay. (II) The survival rate of N2 L4440 (L4440 RNAi N2) and N2 *fbxc-58* (*fbxc-58* RNAi N2) in PA14 infection for 24 h at day 9 of adulthood. All of relative mRNA levels were determined by RT-PCR by three times independent experiments, normalized to *act-3*. Error bars represent SEM. Abbreviation: ns: not significant, ^*^p < 0.05, ^**^p < 0.01, ^***^p < 0.001; unpaired *t* test.

To uncover the innate immune signal that mediates the DR effect, we hypothesized that candidate genes would be upregulated in response to both DR and pathogen infection. Based on this assumption, we analyzed previously reported DEG data for DR [12] and the pathogen [*Pseudomonas aeruginosa* (PA14)]-infected condition [20]. As shown in Figure 1A, we found that 1,085 and 890 genes were upregulated by DR and PA14 infection, respectively. Among these genes, we investigated those (107 genes) that were simultaneously upregulated under both conditions (Figure 1A, Supplementary Table 1) and found that F-box genes (7.5%) fit this criterion (Supplementary Table 1). Among the F-box genes, we found that *fbxc-58* was most highly expressed (~4-fold) under DR conditions (induced by *eat-2* mutation) (Figure 1B). Furthermore, *fbxc-58* expression also increased 2.2-fold under low density of food (LDF) feeding conditions [4] compared with that under *ad libitum* (AL) conditions (Figure 1C), implying that *fbxc-58* was upregulated in response to DR.

Next, we tested whether *fbxc-58* exhibits resistance to pathogen. Note that *fbxc-58* RNAi significantly downregulated *fbxc-58* expression in *C. elegans* (Figure 1D). By treating PA14 to *fbxc-58* RNAi or L4440 RNAi worms, as shown in Figure 1E, we found that the death rate of *fbxc-58* RNAi worms was significantly higher than that of L4440 RNAi worms (*P* < 0.001). This data implying that *fbxc-58* is a pathogen defense-related gene. Thus, *fbxc-58* satisfies the condition to be regarded as an immune signaling component whose expression increases in response to DR. Therefore, we further investigated the role of *fbxc-58* as a mediator of DR effects.

### *fbxc-58* mediates DR-induced lifespan extension in *C. elegans*

**Figure 2:**
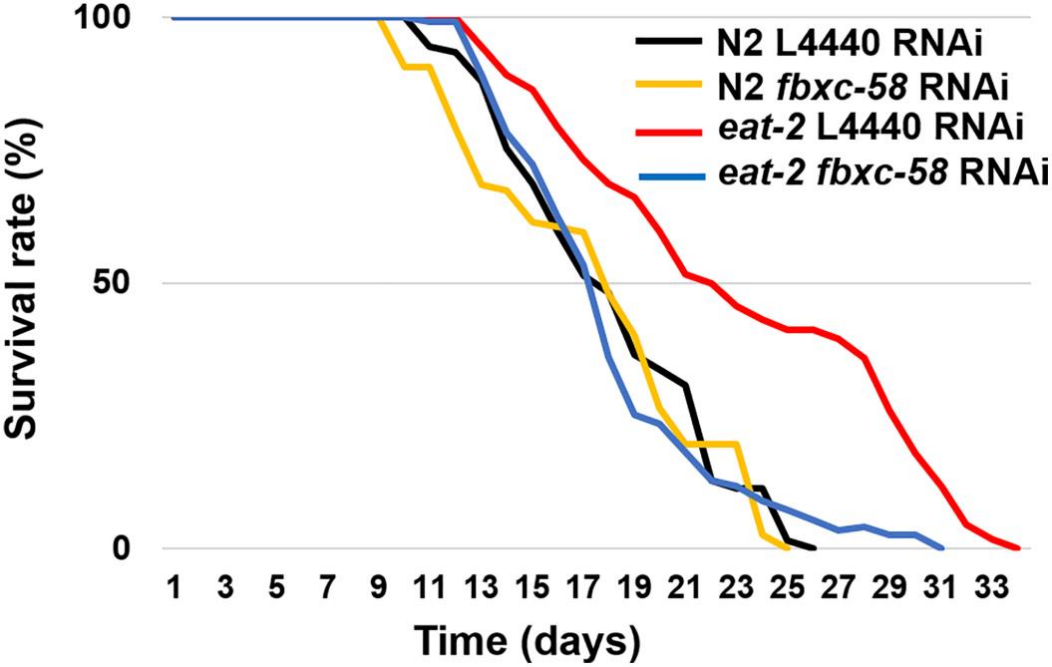
***fbxc-58* mediates dietary restriction effects on longevity.** Survival rate curves of N2 L4440 RNAi (*n* = 105), N2 *fbxc-58* RNAi (*n* = 131), *eat-2* L4440 RNAi (*n* = 112), and *eat-2 fbxc-58* RNAi (*n* = 111). Survival data are summarized in Table 1.

We confirmed that the *eat-2* mutation, which mimic DR, considerably (30.2%) extended the median lifespan (ML) of *C. elegans* [ML of L4440 RNAi N2: 17.87 ± 0.44, ML of L4440 RNAi *eat-2*: 23.27 ± 0.62; *P* < 0.0001] (Figure 2 and Table 1). To determine the effect of *fbxc-58* on longevity in DR, we treated worms with *fbxc-58* RNAi. As shown in Figure 2, we found that the median lifespan of *eat-2(ad1116)* mutants drastically decreased when *fbxc-58* was silenced [ML of L4440 RNAi *eat-2*: 23.27 ± 0.62, ML of *fbxc-58* RNAi *eat-2*: 18.10 ± 0.39; (*P* < 0.0001)]. In addition, the median lifespan of *fbxc-58* RNAi *eat-2(ad1116)* mutants was similar to that of the wild-type strain [ML of *fbxc-58* RNAi N2: 17.99 ± 0.37, ML of *fbxc-58* RNAi *eat-2*: 18.10 ± 0.39; *P* = 0.86] (Figure 2 and Table 1). Notably, silencing *fbxc-58* did not alter the median lifespan of the wild-type strain [ML of L4440 RNAi N2: 17.87 ± 0.44, ML of *fbxc-58* RNAi N2: 17.99 ± 0.37; *P* = 0.89] (Figure 2 and Table 1). Thus, these data imply that *fbxc-58* affects DR, playing a pivotal role in extending longevity.

**Table 1:** Median lifespans of *C. elegans* cohorts.

**Figure**	**Strain**	**RNAi treatment**	**Mean lifespan (ML)**	**No. of worms (No. of experiments)**
Figure 2	N2	L4440 RNAi	17.87 ± 0.44	105 (3)
	N2	*fbxc-58* RNAi	17.99 ± 0.37	131 (3)
	*eat-2*	L4440 RNAi	23.27 ± 0.62	112 (3)
	*eat-2*	*fbxc-58* RNAi	18.10 ± 0.39	111 (3)
Figure 5C	N2	L4440 RNAi	18.06 ± 0.44	90 (2)
	N2	*fbxc-58* RNAi	17.99 ± 0.39	90 (2)
	*rsks-1*	L4440 RNAi	21.84 ± 0.42	81 (2)
	*rsks-1*	*fbxc-58* RNAi	17.32 ± 0.37	68 (2)

### *fbxc-58* delays muscle aging of *C. elegans* in DR

**Figure 3:**
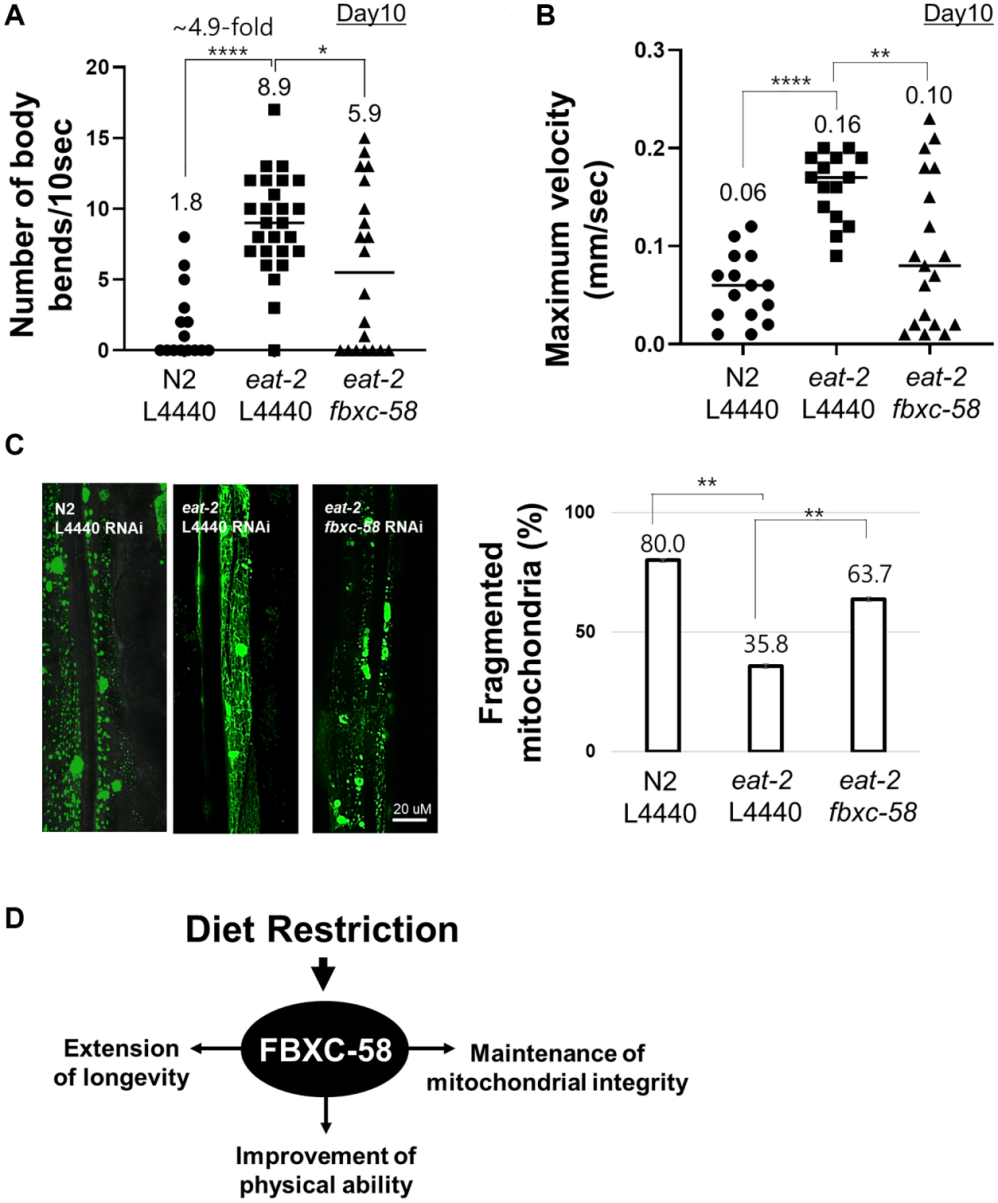
***fbxc-58* mediates dietary restriction effects on mitigating muscle aging.** (**A**) The number of body bends in N2 L4440 RNAi (N2 L4440) (*n* = 15), *eat-2* L4440 RNAi (*eat-2* L4440) (*n* = 26), and *eat-2 fbxc-58* RNAi (*eat-2 fbxc-58*) (*n* = 20) at day 10 of adulthood. (**B**) MVs of N2 L4440 RNAi (N2 L4440) (*n* = 15), *eat-2* L4440 RNAi (*eat-2* L4440) (*n* = 15), and *eat-2 fbxc-58* RNAi (*eat-2 fbxc-58*) (*n* = 19) at day 10 of adulthood. (**C**) The morphological categories of mitochondria are defined as follows. Images showing most of the long interconnected mitochondrial networks were classified as tubular, and images showing most of the short mitochondria or sparse globular mitochondria were classified as fragmented. Mitochondrial morphology was examined in PD4251 and *eat-2(ad1116)*; PD4251 strain that emits fluorescence from mitochondria due to GFP expressed in mitochondria. (Left) Representative images of N2 L4440 RNAi (N2 L4440), *eat-2* L4440 RNAi and *eat-2 fbxc-58* RNAi at day 8 of adulthood. (Right) Qualitative analysis of mitochondrial morphology in N2 L4440 RNAi (N2 L4440), *eat-2* L4440 RNAi (*eat-2* L4440) and *eat-2 fbxc-58* RNAi (*eat-2 fbxc-58*) at day 8 of adulthood. Bars represent the proportion of worms with fragmented mitochondria. (**D**) A schematic diagram of *fbxc-58* mediating DR-induced effects. Error bars represent SEM. ^*^p < 0.05, ^**^p < 0.01, ^****^p < 0.0001; unpaired *t* test.

Aged *eat-2(ad1116)* mutants showed a 4.9-fold (*P* < 0.0001) higher body bending rate than that of the aged N2 strain (Figure 3A, Supplementary Figure 1A), although N2 and *eat-2(ad1116)* mutants showed similar body bending rates in the young adult stage (Supplementary Figure 1B). This data implies that DR alleviates the decline in physical activity of *C. elegans* during aging. Notably, we found that the improved muscle activity of aged *eat-2(ad1116)* mutants was significantly diminished by *fbxc-58* RNAi (*P* < 0.05) (Figure 3A, Supplementary Figure 1), implying that *fbxc-58* is vital to improving muscle activity through DR in *C. elegans*. Note that there was no statistical difference in body bending rates by *fbxc-58* RNAi in aged N2 strains (Supplementary Figure 2).

Further, in *C. elegans*, maximum body movement velocity (MV) is a potent indicator of their health condition and muscle activity [21]. As shown in Figure 3B and Supplementary Figure 3A, the aged *eat-2(ad1116)* mutant strain exhibited higher MV than that of the aged N2 strain, although MV in the young N2 strain was slightly higher than that of young *eat-2(ad1116)* mutants (Supplementary Figure 3B). However, the MV of aged *eat-2(ad1116)* mutants significantly decreased when *fbxc-58* was abrogated (*P* < 0.01) (Figure 3B, Supplementary Figure 3A). This data signifies the essentiality of *fbxc-58* in improving the physical health of aged *C. elegans* through DR. Note that there was no statistical difference in MVs by *fbxc-58* RNAi in aged N2 strains (Supplementary Figure 4).

In aging, the disintegration of mitochondrial networks (e.g., mitochondrial fragmentation) in muscle cells is a well-established phenotype of muscle aging in *C. elegans* [22], and this mitochondrial damage is regarded as one of the leading factors contributing to muscle aging [23]. We found that the proportion of worms with fragmented mitochondria in body wall muscle was significantly lower in L4440 RNAi *eat-2(ad1116)* mutants (35.8%) than that in the L4440 RNAi N2 strain (80.0%). However, this proportion was increased when *fbxc-58* was silenced in *eat-2(ad1116)* mutants (63.7%) (*P* < 0.01) (Figure 3C), implying that *fbxc-58* plays a pivotal role in maintaining the mitochondrial networks in muscle cells, and it is consistent with the reduced muscle activity in *fbxc-58* RNAi *eat-2(ad1116)* mutants relative to that in L4440 RNAi *eat-2(ad1116)* mutants (Figure 3A, 3B). Note that *fbxc-58* RNAi did not affect the mitochondrial morphology of aged N2 strains (Supplementary Figure 5). Thus, *fbxc-58* is necessary for DR-induced longevity extension, muscle mitochondrial protection, and physical activity improvement in aging (Figure 3D).

### *fbxc-58* is a downstream target of ZIP-2 and PHA-4

**Figure 4:**
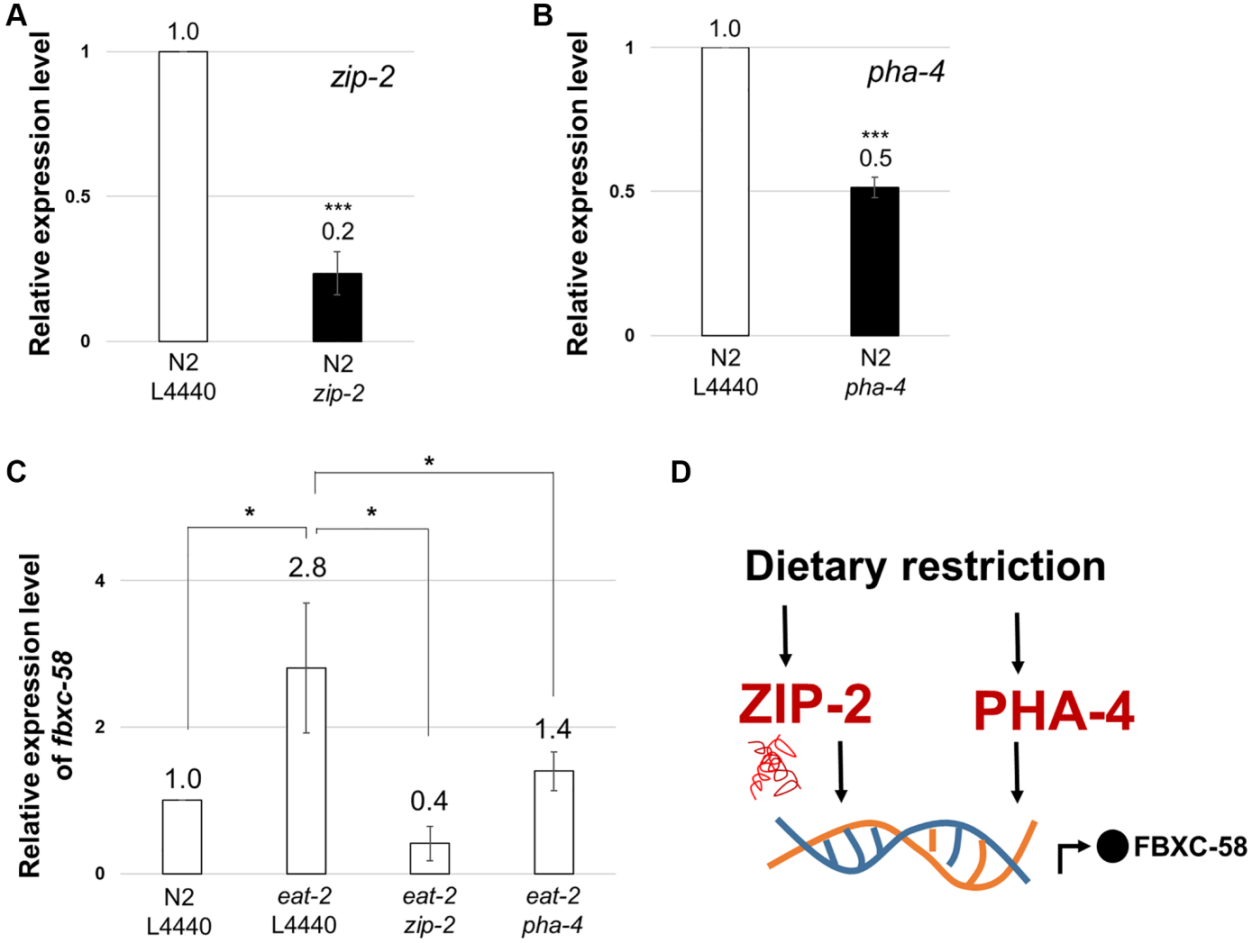
***fbxc-58* is downstream target of ZIP-2 and PHA-4.** (**A**) Relative expression levels of *zip-2* mRNA in N2 L4440 RNAi (N2 L4440) and N2 *zip-2* RNAi (N2 *zip-2*). (**B**) Relative expression levels of *pha-4* mRNA in N2 L4440 RNAi (N2 L4440) and N2 *pha-4* RNAi (N2 *pha-4*). (**C**) Relative expression level of *fbxc-58* in N2 L4440 RNAi (N2 L4440), *eat-2* L4440 RNAi (*eat-2* L4440), *eat-2 zip-2* RNAi (*eat-2 zip-2*), and *eat-2 pha-4* RNAi (*eat-2 pha-4*) at day 3 of adulthood. (**D**) A schematic diagram of *fbxc-58* induction mechanism in DR through ZIP-2 and PHA-4. All of relative mRNA levels were determined by RT-PCR by three times independent experiments, normalized to *act-3*. Error bars represent SEM. Abbreviation: ns: not significant, ^*^p < 0.05, ^***^p < 0.001; unpaired *t* test.

To understand the underlying molecular mechanism of *fbxc-58* in DR in detail, we examined the correlation between *fbxc-58* and *zip-2* since ZIP-2 is a well-known immune response effector in *C. elegans* [24]. Additionally, ZIP-2 is a transcription factor that regulates aging process [25] and the extent of longevity under various DR conditions, including dietary deprivation, diluted peptone, or diluted OP50 without peptone [4]. Note that we confirmed that *zip-2* or *pha-4* significantly reduced under *zip-2* RNAi or *pha-4* RNAi conditions, respectively (Figure 4A, 4B). As shown in Figure 4C, we found that *fbxc-58* expression was significantly diminished by silencing *zip-2* in *eat-2(ad1116)* mutants compared with that in the L4440 RNAi control. This data implies that *fbxc-58* is upregulated by *zip-2* under DR conditions. Additionally, we confirmed that C10C5.2 (sequence name of *fbxc-58*) is among the genes that are downregulated by *zip-2* RNAi in previously reported differentially expressed gene (DEG) data [24], indicating that *fbxc-58* is a downstream target gene of ZIP-2.

PHA-4 is the forkhead box transcription factor that mediates the lifespan extension induced by *eat-2* mutation [26]. We found that *fbxc-58* expression was decreased in *pha-4* RNAi *eat-2(ad1116)* mutants compared with that in the L4440 RNAi *eat-2(ad1116)* mutant strains (Figure 4C). This data implies that *fbxc-58* is a downstream target gene of PHA-4. Thus, all of these data corroborate the fact that *fbxc-58* is indeed a downstream effector of DR (Figure 4D).

### *fbxc-58* is a downstream effector of S6 kinase pathway

**Figure 5:**
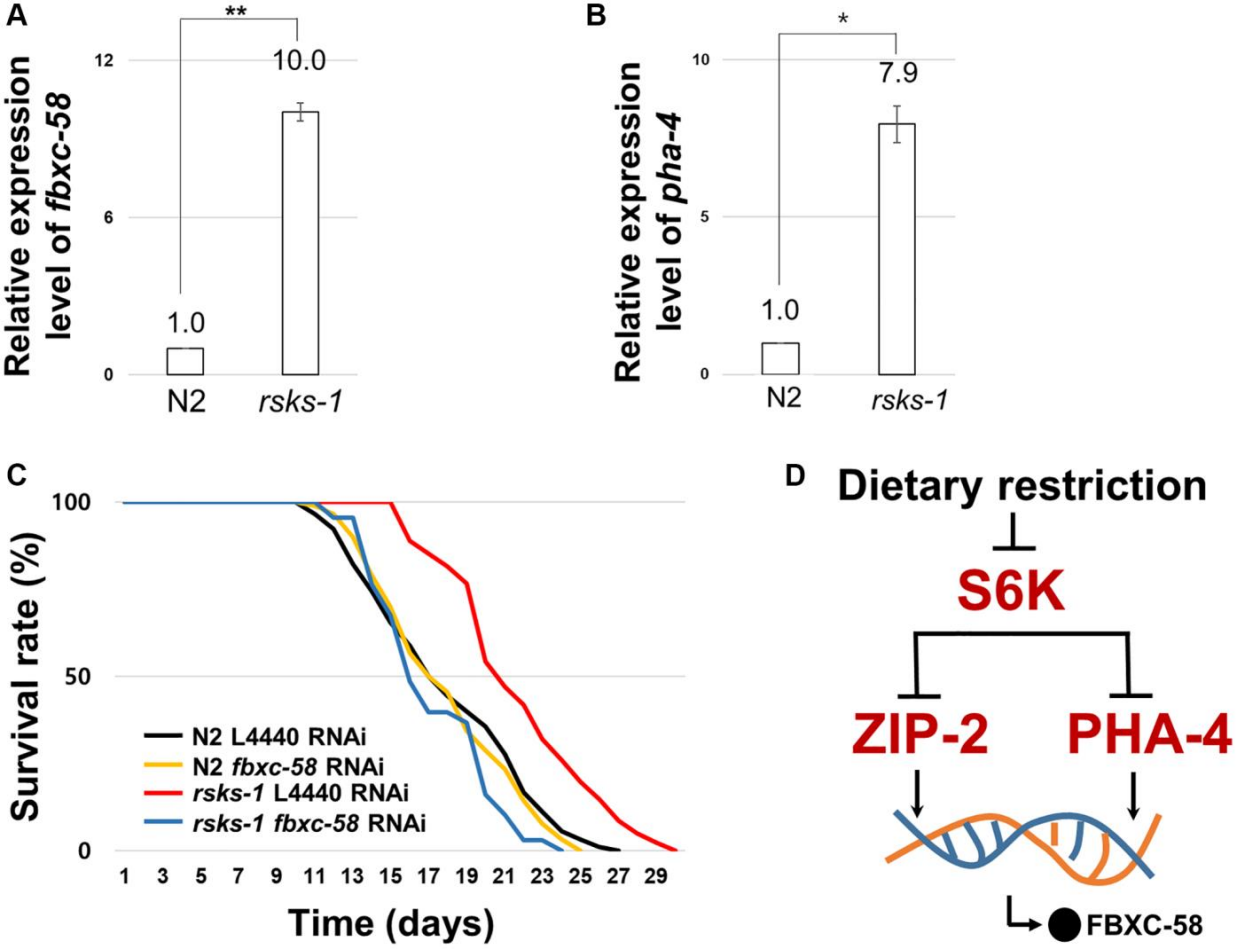
***fbxc-58* is downstream effector of S6Kinase signaling pathway.** (**A**) Relative expression level of *fbxc-58* in N2 and *rsks-1(tm1714)* mutant strains. (**B**) Relative expression level of *pha-4* in N2 and *rsks-1(tm1714)* mutant strains. (**C**) Survival rate curves of N2 L4440 RNAi (*n* = 90), N2 *fbxc-58* RNAi (*n* = 90), *rsks-1* L4440 RNAi (*n* = 81), and *rsks-1 fbxc-58* RNAi (*n* = 68). Survival data are summarized in Table 1. (**D**) A schematic diagram of *fbxc-58* induction mechanism in DR through S6K, ZIP-2, and PHA-4. All of relative mRNA levels were determined by RT-PCR by three times independent experiments, normalized to *act-3*. Error bars mean SEM. ^*^p < 0.05, ^**^p < 0.01; unpaired *t* test.

Mutation in S6 kinase inhibits its translation [27], mediating lifespan extension through DR [28]. *zip-2* is necessary for the lifespan extension of the *rsks-1* (*C. elegans* homolog of S6 kinase) mutant strain [4]. Further, PHA-4 is a downstream effector of S6K pathway for longevity in *C. elegans* [29]. Thus, we tested whether *fbxc-58* is necessary for lifespan extension by *rsks-1* mutation. We found that *fbxc-58* expression was increased in *rsks-1(tm1714)* mutants compared with that in the N2 strain (Figure 5A). Note that the positive control gene, *pha-4* [29], was also increased in *rsks-1(tm1714)* mutants (Figure 5B). Further, as shown in Figure 5C and Table 1, we observed that *rsks-1(tm1714)* mutant strains showed elongated ML compared to N2 [ML of L4440 RNAi N2: 18.06 ± 0.44, ML of L4440 RNAi *rsks-1*: 21.84 ± 0.42; (*P* < 0.0001)]. In addition, *fbxc-58* RNAi significantly reduced the lifespan of *rsks-1(tm1714)* mutants [ML of L4440 RNAi *rsks-1*: 21.84 ± 0.42, ML of *fbxc-58* RNAi *rsks-1*: 17.32 ± 0.37; (*P* < 0.0001)], indicating that *fbxc-58* is necessary for extending the longevity of *rsks-1(tm1714)* mutants, and this implies that *fbxc-58*-mediated lifespan extension in DR is dependent of S6 kinase signaling pathway (Figure 5D).

## DISCUSSION

DR is a practically effective and reproducible nutritional intervention that extends lifespan in many organisms. However, the underlying mechanisms mediating the effects of DR remain largely unknown. Many studies have shown that DR improves immune function, and immune signaling components are required for DR-induced lifespan extension. These results support the idea that the immune system acts as an important mechanism for DR-induced longevity. Recently, analysis of genes that regulate aging or immune response in animal models, including *C. elegans*, *Drosophila*, mice, and even humans, has revealed that aging and immunity are controlled by the same signaling pathways, such as TOR/S6K signaling pathway, pleiotropically [30]. DR-induced longevity is also associated with the modulation of the TOR/S6K signaling pathway [31–33]. Thus, these results suggest that the immune function may be closely associated with aging regulation through DR. In this study, we found that the F-box gene *fbxc-58* is a downstream effector of the S6K signaling pathway (Figure 5), and that it regulates both pathogen resistance and aging in *C. elegans*. Furthermore, *fbxc-58* is necessary for the effects of DR on lifespan extension. F-box protein contains conserved F-box domain [19], which acts as a site for protein-protein interaction. F-box protein acts as a modular E3 ubiquitin ligase adaptor protein, and the ubiquitin-dependent mechanisms have been shown to determine lifespan in response to DR [34] or modulate the innate immune response [35]. Therefore, we suggest that gaining insights into the detailed mechanistic aspects of *fbxc-58* signaling pathway could elucidate the conserved signaling mechanism that links innate immunity and DR-induced healthy aging in animals.

Further, DR prevents or reduces the burden of age-related diseases or disabilities. Especially, in an aging and sedentary society, sarcopenia, an age-associated muscle disease, is beginning to be recognized as an acute disease condition. Although an effective sarcopenia treatment regime has not yet been identified, nutritional intervention is considered an effective method of preventing sarcopenia [36]. In this study, we found that DR prevents muscle aging via *fbxc-58* in *C. elegans*. *fbxc-58* is essential for DR-mediated alleviation of the age-associated decline in muscle activity and protection of mitochondrial network in body wall muscle. Thus, we propose that investigating the molecular mechanism of action of F-box proteins, including *fbxc-58,* in DR will shed light on means to prevent sarcopenia and offer a potentially practical means of encouraging healthy aging via DR.

## MATERIALS AND METHODS

### Strains

The following strains were used. N2 wild-type, *eat-2(ad1116)*, *rsks-1(tm1714)*, PD4251 ccIs4251 [(pSAK2) *myo-3*p::GFP::LacZ::NLS + (pSAK4) *myo-3*p::mitochondrial GFP + *dpy-20*(+)]; *dyp-20(e1282)*, and *eat-2(ad1116)*; PD4251. In this study, all strains were maintained at 20°C.

### Quantitative-RT PCR

Total RNA was extracted by using RNeasy Fibrous Tissue Mini Kit (Qiagen, Cat No. 79306). cDNA was generated by using a reverse transcription system (ReverTra Ace^™^ qPCR RT Master Mix, Toyobo, Cat No. FSQ-201) and was used for quantitative PCR. Quantitative real time PCR was performed with SYBR^®^ Green Realtime PCR Master Mi (Toyobo, Cat No. QPK-201) using A ViiA 7 Real-Time PCR System (ThermoFisher) and analyzed using ΔΔCt methods described in the manufacturer’s manual. Sequences of primers used for quantitative RT-PCR analysis; *fbxc-37*-Forward: AGTTCGAGATGATCTGCTGC, and *fbxc-37*-Reverse: TCCAATTGTTAACCACCCATG, *fbxc-63*-Forward: GATTTTCCAAGCCAAGTCGC, *fbxc-63*-Reverse: ACTACCACCAGTATAGTCCG, *fbxc-69*-Forward: GATGAATTATCCGTGCAAACTG, *fbxc-69*-Reverse: TCACATCACCATTGTAATCCG, *fbxc-79*-Forward: GATGCTAGGAAGATTGAAGAG, *fbxc-79*-Reverse: CCATACTGTACTTCAAACGAG, *fbxc-83*-Forward: GCTCACGTTCACAGAAGAAG, *fbxc-83*-Reverse: AAAACTGCGTTGTTGGCTTTG, *fbxc-98*-Forward: GCAAAAGTTAGAGATGTGCTC, *fbxc-98*-Reverse: CAGTAGAATGCACACGTAATG, *fbxc-58*-Forward: CAGAAGAGGAGAAACCGAAG, and *fbxc-58*-Reverse: GCAAGGAGTCTCACTCTTTTC, *pha-4*-Forward: CTGTTAATCACAGTCAACCTAC, *pha-4*-Reverse: GTGTTGTTCAGGAAATTCTGG, *irg-1*-Forward: GCTGAAATTCACTTGTAGTGAG, *irg-1*-Reverse: GAGACCATAATTTCAATTGCTC, *act-3*-Forward: AAGTCATCACCGTCGGAAAC, and *act-3*-Reverse: TTCCTGGGTACATGGTGGTT. All qRT-PCR experiments were performed as three independent experiments.

### Low density food (LDF) feeding experiments

To test DR effect by limiting food concentration, the nematode growth medium (NGM) plate without peptone was used for limiting the density of food (OP50 *E. coli*). After culturing the OP50 strain in Luria-Bertani (LB) broth containing streptomycin (20 mg/mL) at 37°C overnight, the culture mixture was centrifuged, and the pellets were resuspended and washed with M9 buffer containing streptomycin. The concentration of the OP50 on the assay plates was determined using a UV spectrometer, and the optical density (OD) value of bacteria in the LDF condition is 0.36. 200 μL aliquots of LDF (0.36 OD) preparation was spread over the assay plate. Synchronized worms were transferred to NGM (ad libitum, AL) or LDF plates at the young adult stage. After 48 hours, worms were collected and used for RT-qPCR experiments to observe the expression level of *fbxc-58* (Figure 1C).

### Life span analysis

The number of live animals was scored every 1–2 day until death in solid NGM plate. The death of worm was defined as the failure to respond to gentle prodding on the head or tail with a platinum wire. Life span was assessed at 20°C, and assay was performed without FUDR treatment. Life span was analyzed by Oasis survival analysis software (https://sbi.postech.ac.kr/oasis/) [37].

### Qualitative analysis of mitochondrial morphology

The morphological categories of mitochondria are defined as follows. Images showing most of the long interconnected mitochondrial networks were classified as tubular, and images showing most of the short mitochondria or sparse globular mitochondria were classified as fragmented. Mitochondrial morphology was examined in *eat-2(ad1116)*; PD4251 strain that emits fluorescence from mitochondria due to GFP expressed in mitochondria. Worms were immobilized during imaging using 100 mM sodium azide. Imaging was performed using a microscope equipped with an Olympus Fluoview FV3000 confocal laser scanning microscope. FV3000 RS Fluoview software was used to acquire fluorescent z stacks of individual animals (1 μm/slice).

### Measurement of worm’s body bend number

The body bend number of worms tested in liquid M9 buffer. The number of changes in the reciprocating motion of bending at the center of the body was counted. The body bend was observed for 10 seconds by an Olympus SZX7 zoom stereo microscope (Olympus Corporation, Japan).

### Measurement of worm’s maximum velocity (MV)

The physical assay plate was NGM plate without peptone and with no bacterial lawn. Synchronized single worms were transferred to the physical assay plate and movements recorded immediately. The recording system comprised a stereomicroscope (Olympus SZX7), a CCD camera (Olympus DR74), and imaging software (TUCSEN ISCapture). The locomotion velocity was expressed as mm per second (the distance (mm) between displaced centroids per second). Recorded images were analyzed by ImageJ and wrMTrck (plugin for ImageJ: https://www.phage.dk/plugins). The locomotion velocity data were imported into an Excel spreadsheet, and the peak locomotion velocity in the 30 s period was used as the MV.

### RNAi experiments

In this study, commercial *C. elegans* RNAi feeding libraries generated by the Ahringer laboratory (Geneservice Ltd., Cambridge, UK) was used. For gene expression observation, RNAi treatment was for 72 hours from young adulthood. In addition, each young adult worms were transferred to each RNAi plate and life span (Figure 2, Figure 5C) or physical activities (number of body bends or MV) (Figure 3A, 3B and Supplementary Figures 1–4) or mitochondrial morphology (Figure 3C and Supplementary Figure 5) were examined.

### Data availability statement

The data that support the findings of this study are available from the corresponding author upon reasonable request.

## Supplementary Materials

Supplementary Figures

Supplementary Table 1
