# Supplementary material for: The innate immune signaling component FBXC-58 mediates dietary restriction effects on healthy aging in Caenorhabditis elegans
Source: Aging (Albany NY). 2023 Jan 6;15(1):21–36. doi: 10.18632/aging.204477 (PMC9876644; doi:10.18632/aging.204477)
Supplement: Supplementary Figures [file aging-15-204477-s001.pdf]

SUPPLEMENTARY FIGURES

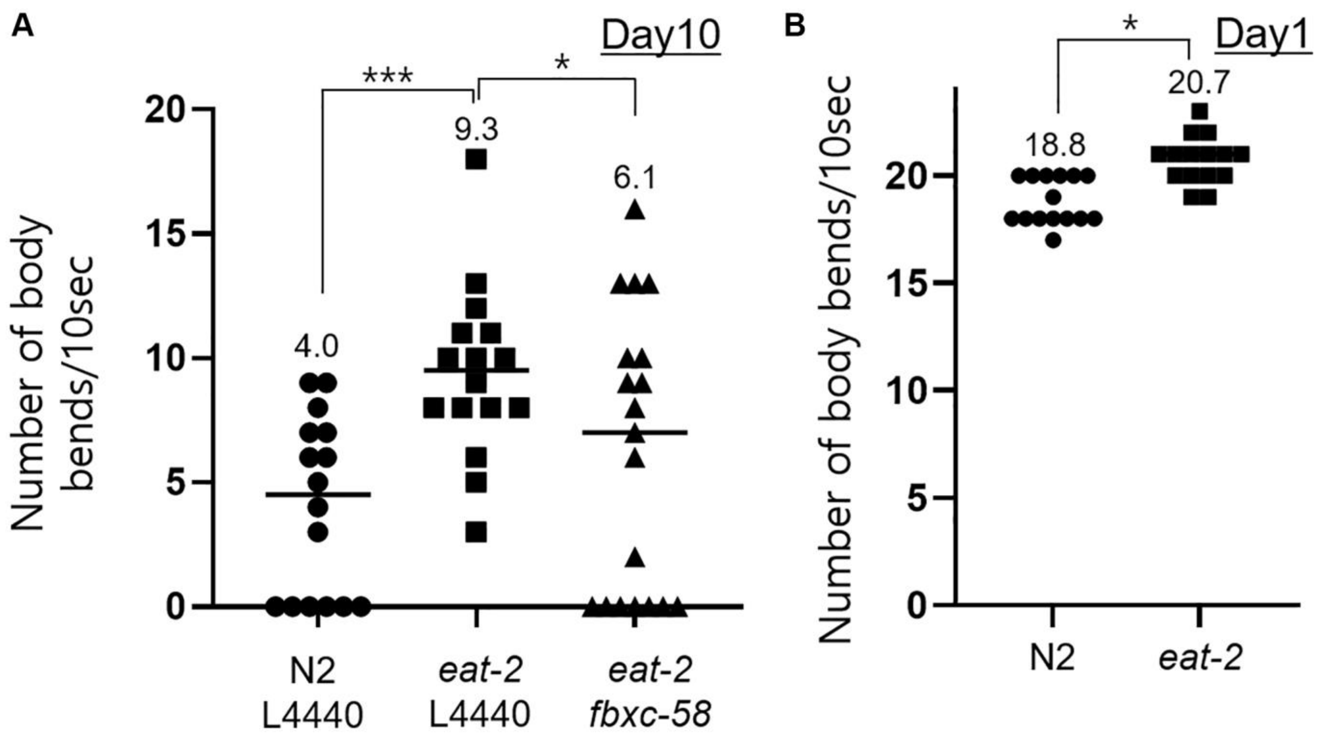

Supplementary Figure 1. The number of body bends of worms. (**A**) The number of body bends in N2 L4440 RNAi (N2 L4440) (n = 16), *eat-2* L4440 RNAi (*eat-2* L4440) (n = 16), and *eat-2 fbxc-58* RNAi (*eat-2 fbxc-58*) (n = 19) at day 10 of adulthood. (**B**) The number of body bends in N2 (n = 31) and *eat-2* mutant strains (n = 30) at day 1 of adulthood. $^{*}p < 0.05$, $^{***}p < 0.001$; unpaired t test.

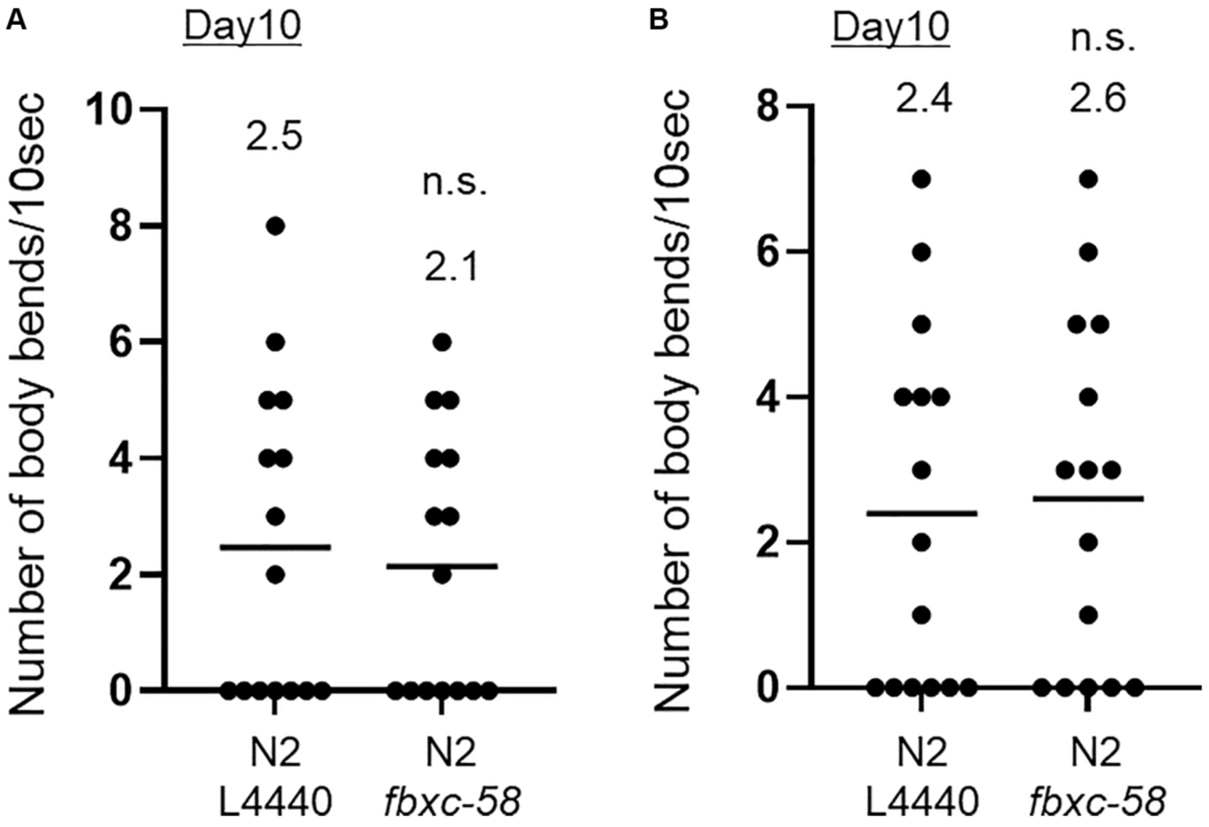

Supplementary Figure 2. The number of body bends of worms. Two independent experiments. (**A**) The number of body bends in N2 L4440 RNAi (N2 L4440) (n = 15), N2 *fbxc-58* RNAi (N2 *fbxc-58*) (n = 15) at day 10 of adulthood. (**B**) The number of body bends in N2 L4440 RNAi (N2 L4440) (n = 15), N2 *fbxc-58* RNAi (N2 *fbxc-58*) (n = 15) at day 10 of adulthood. Abbreviation: n.s: not significant; unpaired t test.

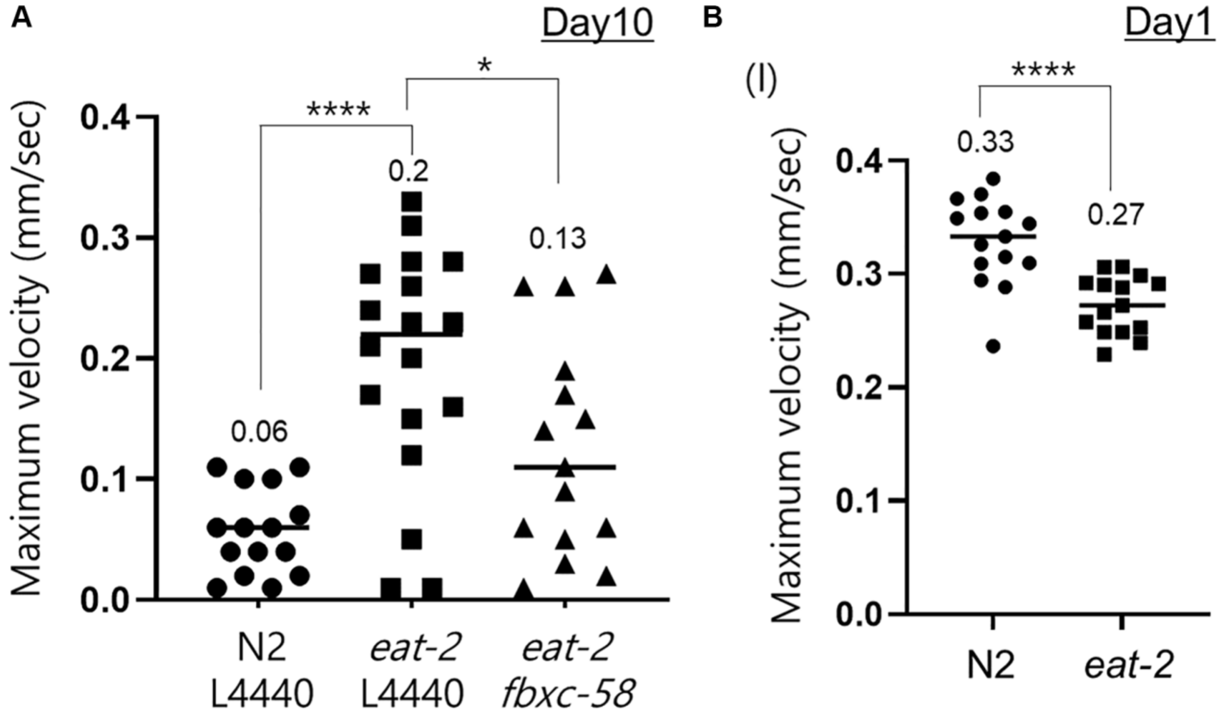

Supplementary Figure 3. Maximum velocity (MV) of worms. (**A**) MVs in N2 L4440 RNAi (N2 L4440) (n = 15), *eat-2* L4440 RNAi (*eat-2* L4440) (n = 18), and *eat-2 fbxc-58* RNAi (*eat-2 fbxc-58*) (n = 15) at day 10 of adulthood. (**B**) MVs in N2 (n = 15) and *eat-2* mutant strains (n = 15) at day 1 of adulthood. $^{*}p < 0.05$, $^{****}p < 0.0001$; unpaired t test.

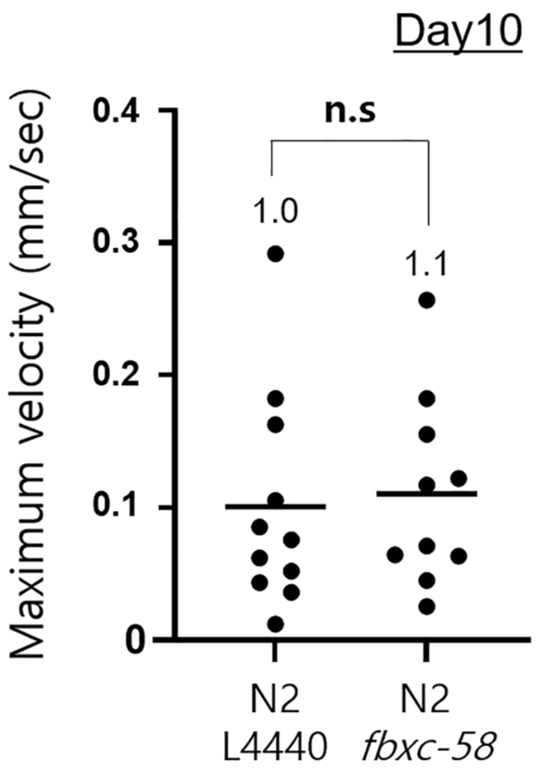

Supplementary Figure 4. Maximum velocity (MV) of worms. MVs in N2 L4440 RNAi (N2 L4440) (n = 11) and N2 *fbxc-58* RNAi (N2 *fbxc-58*) (n = 10) at day 10 of adulthood. Abbreviation: n.s: not significant; unpaired t test.

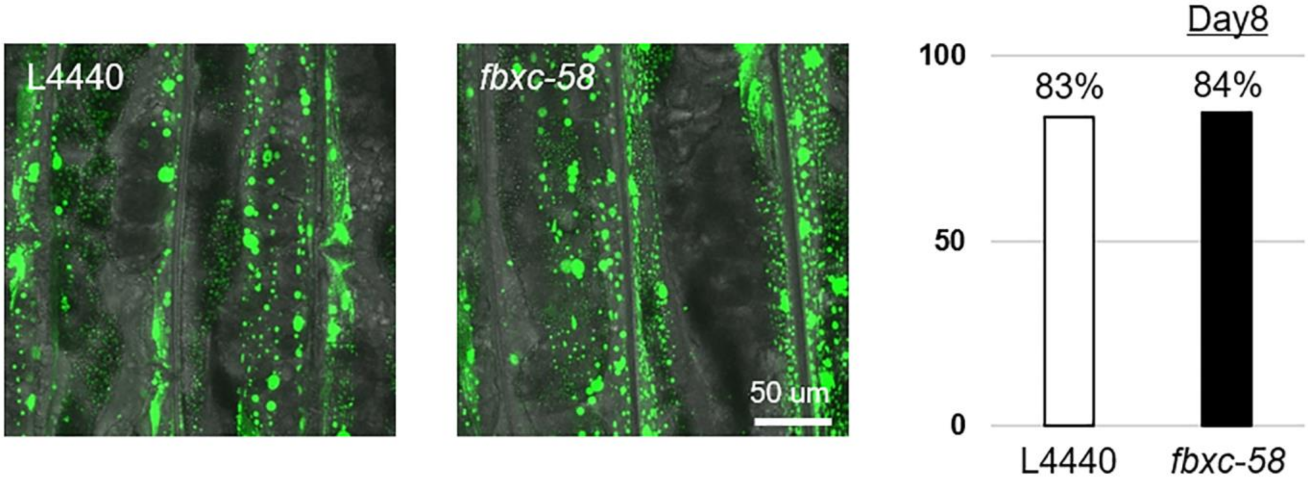

Supplementary Figure 5. Mitochondrial morphology. (Left) Representative images of N2 L4440 RNAi (L4440) and N2 *fbxc-58* RNAi at day 8 of adulthood. (Right) Qualitative analysis of mitochondrial morphology in N2 L4440 RNAi (L4440) (n = 12) and N2 *fbxc-58* RNAi (n = 13) at day 8 of adulthood. Bars represent the proportion of worms with fragmented mitochondria. Mitochondrial morphology was examined in PD4251 that emits fluorescence from mitochondria due to GFP expressed in mitochondria.
