# Supplementary material for: The innate immune signaling component FBXC-58 mediates dietary restriction effects on healthy aging in Caenorhabditis elegans
Source: Aging (Albany NY). 2023 Jan 6;15(1):21–36. doi: 10.18632/aging.204477 (PMC9876644; doi:10.18632/aging.204477)
Supplement: Supplementary Table 1 [file aging-15-204477-s002.pdf]

SUPPLEMENTARY TABLE

Supplementary Table 1. Genes simultaneously upregulated in both DR and pathogen infected conditions.

Number	Gene or sequence name	Number	Gene or sequence name
1	*asp-12*	55	*fbxa-98*
2	*B0416.7*	56	*gem-4*
3	*best-24*	57	*gst-16*
4	*C04A11.5*	58	*H02F09.3*
5	*C05D9.9*	59	*H32K16.2*
6	*C10A4.10*	60	*hil-1*
7	*C10C5.2 (fbxc-58)*	61	*irg-2*
8	*C16E9.1*	62	*lgc-47*
9	*C17B7.4*	63	*lipl-1*
10	*C18A11.1*	64	*M01G12.9*
11	*C25F9.11*	65	*M04G7.3*
12	*C25F9.12*	66	*M60.2*
13	*C29F9.4*	67	*nas-5*
14	*C34C6.7*	68	*nlp-5*
15	*C35C5.8*	69	*oac-7*
16	*C42D4.18*	70	*pan-1*
17	*C45B11.6*	71	*pgp-6*
18	*C49C8.8*	72	*pgp-7*
19	*C49F5.7*	73	*pud-1.2*
20	*C49G7.12*	74	*pud-2.1*
21	*C49G7.7*	75	*pud-2.2*
22	*C50F4.1*	76	*pud-3*
23	*C50F7.5*	77	*R05D8.7*
24	*C53B7.3*	78	*R05G6.10*
25	*C54D10.14*	79	*R05H10.1*
26	*crn-2*	80	*sdz-35*
27	*cutl-16*	81	*sodh-1*
28	*cyp-37B1*	82	*spp-18*
29	*dhrs-4*	83	*sptf-2*
30	*E03H4.8*	84	*srw-86*
31	*ech-9*	85	*T03F1.6*
32	*elt-3*	86	*T04C12.3*
33	*F08G2.5*	87	*T05F1.9*
34	*F08G5.3*	88	*T16G1.4*
35	*F11D11.3*	89	*T16G1.5*
36	*F14F9.4*	90	*T24A6.7*
37	*F16H6.10*	91	*T24B8.3*
38	*F18E9.3*	92	*tir-1*

39	***F21C10.10***	93	***ugt-18***
40	***F21C10.11***	94	***ugt-24***
41	***F32A5.8***	95	***ugt-29***
42	***F42C5.4***	96	***W04A8.4***
43	***F43C11.7***	97	***Y17D7B.2***
44	***F46C5.1***	98	***Y43C5A.3***
45	***F52B11.5***	99	***Y45F10D.6***
46	***F54C8.6***	100	***Y48G8AL.13***
47	***F56C4.4***	101	***Y54G2A.10***
48	***F56D2.5***	102	***Y58A7A.3***
49	***fbxa-33***	103	***Y58A7A.4***
50	***fbxa-37***	104	***Y71G12B.6***
51	***fbxa-63***	105	***Y97E10AR.1***
52	***fbxa-69***	106	***ZC190.4***
53	***fbxa-79***	107	***ZK287.9***
54	***fbxa-83***		
